# Double Burden of Malnutrition (DBM) and Anaemia under the Same Roof: A Bangladesh Perspective

**DOI:** 10.3390/medsci7020020

**Published:** 2019-01-28

**Authors:** Sumaiya Mamun, Christopher Guy Nicholas Mascie-Taylor

**Affiliations:** 1 Institute of Nutrition and Food Science, University of Dhaka, Dhaka 1000, Bangladesh; 2 Department of Archaeology and Anthropology and Department of Public Health and Primary Care, University of Cambridge, Cambridge CB1 8RN, UK; nmt1@cam.ac.uk

**Keywords:** double burden of malnutrition, anaemia, undernutrition, overweight, obesity

## Abstract

The double burden of malnutrition (DBM) and anaemia is a growing concern in developing countries. Using the cross-sectional Bangladesh Demographic Health Survey, 2011, 5763 mother–child pairs were examined. In households where the mother was overweight, 24.5% of children were stunted, 19.8% underweight, 9.3% wasted, and 51.7% anaemic. Significant regional differences were found in DBM and anaemia as well as drinking water source, while DBM alone was more common in more well-off households (based on wealth index) and where the father was employed in skilled or service occupations. More policy and awareness programmes are needed to address the coexistence of child undernutrition and maternal overweight/obesity and anaemia in the same household.

## 1. Introduction

The number of undernourished people in the world increased from 777 million to an estimated 815 million in 2016. At the same time, adult obesity continues to rise in all regions. Multiple forms of malnutrition therefore co-exist, with countries experiencing simultaneously high rates of child undernutrition and adult obesity [1]. Maternal and child double burden (MCDB) can be defined as an overweight mother paired with an undernourished child. Nutrition disparities are more evident in low- and middle-income countries (LMICs) with higher prevalence of undernutrition, overweight or obesity (overnutrition), or both [2,3]. The social determinants of health (SDoH) have been consistently associated with both undernutrition in children and overweight in mothers [4,5,6]. The overall anaemia prevalence among children less than 5 years old is 54.2% in 52 low, lower-middle, and upper-middle countries according to the Demographic and Health Survey (DHS) data, collected between 2005 and 2016 [7].

**Figure 1:**
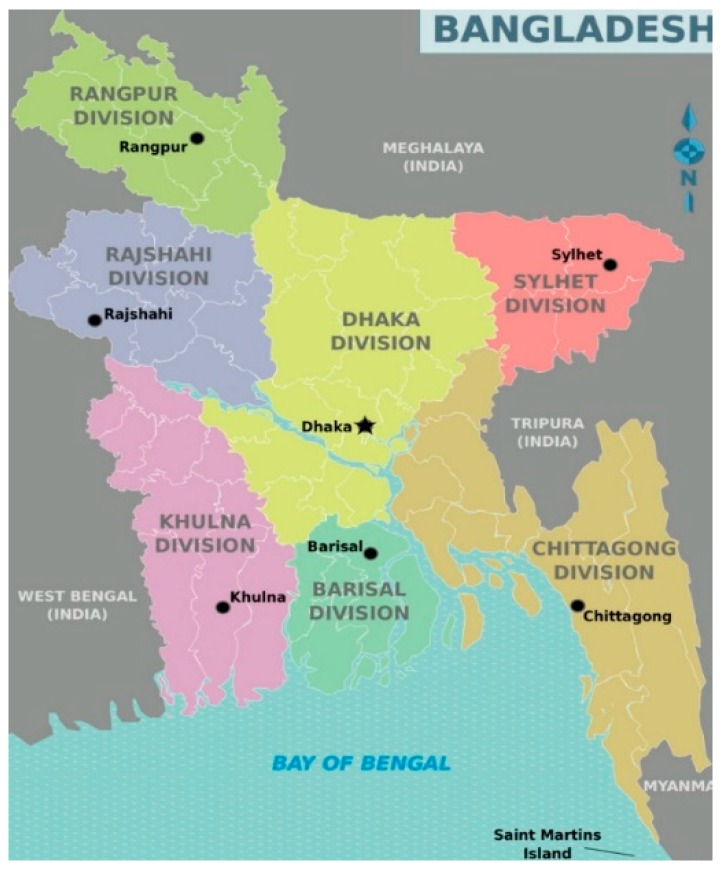
A map of Bangladesh showing seven administrative divisions [21].

In Bangladesh (Figure 1), the prevalence of child undernutrition decreased from 51% to 36% between 2004 and 2007 due to economic growth. On the other hand, maternal overweight increased from 9% to 24% over the same time period [8]. The association between socio-demographic factors and the double burden of malnutrition (DBM) has been examined in several studies [9,10,11,12,13,14,15,16,17,18,19] around the world. In Guatemala, stunted child and overweight mother (SCOM) was more prevalent among poor and middle socioeconomic status groups as compared to the rich households [9]. In Latin America, Mexico, Brazil, Columbia, and Africa, poor maternal education and low socioeconomic status were associated with DBM [10,11,12,13]. On the other hand, studies in China, Indonesia, Vietnam, India, and Bangladesh showed that DBM is more prevalent among high-income households compared to low-income households [14,15,16,17,18]. Jehn et al. used Demographic and Health Survey datasets from 18 lower- and middle-income countries and found that lower level of maternal education, number of siblings, and relative household poverty were associated with DBM [19]. In Bangladesh, only a few small-scale community-based studies have been undertaken with regard to DBM, and no nationwide studies have been undertaken [20].

The present study uses a nationally representative sample from Bangladesh to examine the coexistence of overweight mothers with undernourished (stunted, underweight, and wasted) and anaemic children living in the same household. The study also examines the differences in DBM and anaemia in relation to a number of socio-economic variables. This study provides important information on the reality of the health of the mother and the child in a developing country such as Bangladesh.

## 2. Materials and Methods

The data source for this research was the Bangladesh Demographic and Health Survey (BDHS), 2011. The DHS programmes are nationally representative, cross-sectional household surveys. They provide a wide range of information mainly related to population, health, and nutrition. The sample design used in DHS surveys is mainly based on a two-stage cluster format. In the first stage, enumeration areas (EAs) are generally drawn up from census information. Secondly, a sample household is drawn from each selected EA. Demographic and Health Survey data are representative at the national level, residence level, and regional level [9].

BDHS 2011 obtained informed consent from the participants before the survey. The contents of the household and individual questionnaires were based on the Monitoring and Evaluation to Assess and Use Results (MEASURE) DHS model questionnaires. Ethical permission was obtained by the Macro International Inc. Institutional Review Board on 22 February 2011 when the DHS were carried out, with ICF Macro Project Number: 631561.0.000.00.091.01. All data in the present study were anonymous and no additional ethical permission was required. BDHS used five types of questionnaires: a Household Questionnaire, a Woman’s Questionnaire, a Man’s Questionnaire, a Community Questionnaire, and two Verbal Autopsy Questionnaires to collect data on causes of death among children under age 5. These model questionnaires were adapted for the use in Bangladesh during a series of meetings with a Technical Working Group (TWG) that consisted of representatives from National Institute of Population Research and Training (NIPORT); Mitra and Associates; International Centre for Diarrheal Diseases and Control, Bangladesh (ICDDR,B); United States Agency for International Development (USAID)/Bangladesh; and MEASURE DHS. Draft questionnaires were then circulated to other interested groups and were reviewed by the 2011 BDHS Technical Review Committee [9]. Fieldwork for the 2011 BDHS was carried out by 16 interviewing teams, each consisting of one supervisor, one field editor, five female interviewers, two male interviewers, and one logistics staff member. Data collection was implemented in five phases, starting on 8 July 2011 and ending on 27 December 2011. In addition, during 2–19 January 2012 there were re-visits to collect blood samples from respondents interviewed during Ramadan who had agreed to participate in blood testing but declined to be tested during Ramadan. Fieldwork was also monitored through visits by representatives from USAID, Inner City Fund (ICF) International, and NIPORT [9].

A total of 18,222 ever-married women aged 12–49 were identified in these households, and 17,842 were interviewed, yielding a response rate of 98 percent. In one-third of the households, ever-married men over age 15 were eligible for interview. Because there were only 90 ever-married women aged 12–14 (less than one percent), these women were excluded. Children aged from 0 to 59 months were selected. Only the first child of each family was selected. Only children and their mothers having anthropometric information (height and weight) and all the socio-economic variables were included. Finally, 5783 mother–child pairs were selected based on the abovementioned criteria. The principal reason for nonresponse among women and men was their absence from home despite repeated visits to the household. The response rates do not vary notably by urban–rural residence.

Following WHO recommendations, any children with height-for-age Z-score (HAZ) and weight-for-age Z-score (WAZ) either above +6 or below −6 and Weight for Height Z-score WHZ above +5 or below −5 were excluded. For women, anthropometric measurements above and below 4 standard deviation (SD) were considered outliers and were excluded from the data [22]. Children and women lacking information for any of the socio-economic variables of the study were also excluded. In total, 5763 mother–child pairs with complete information were analysed. The total number of mother–child pairs having haemoglobin data was 1661. The smaller sample size for haemoglobin concentration data was because BDHS chose to only take a sample of blood from every third household. No significant differences were found between mother–child pairs with complete or incomplete information.

The nutritional status was assessed using anthropometry (body mass index (BMI) in mothers and stunting, underweight, and wasting in children) and anaemia status of mother–child pairs. Height and weight were measured using standard procedures by trained staff. Children younger than 2 years were measured lying down (recumbent length) on an anthropometric board, while standing height was measured for older children and mothers. For weighing of children and mothers, a weighing scale was used which allows for weighing of very young children through an automated mother–child adjustment that eliminated the mother’s weight while she was standing on the scale with her baby. Haemoglobin concentration was determined using a Hemocue (Radiometer Medical ApS, Copenhagen, Denmark), a portable photometer which uses the absorption of light from a single drop of blood to measure haemoglobin concentration. Maternal BMI was categorised into three categories of underweight (BMI < 18.5), normal (18.5 to 24.9), and overweight (≥25.0) and stunting, underweight, and wasting by z-scores of <−2.00 of height-for-age, weight-for-age, and weight-for-height, respectively, following the WHO guidelines [22]. All mother–child combinations, e.g., of BMI and stunted and not stunted children, were included in the analyses, but only data on overweight mothers with undernourished children are presented in the results.

χ^2^ testing and sequential multinomial regression analysis was performed to examine the association between maternal and child nutritional status and the socio-economic variables. Initially the analyses removed the linear and quadratic effects of age and sex before testing for the significance of a socio-economic variable. Later, after removing the linear and quadratic effects of age and sex, all the socio-economic variables were entered into the model except for the variable of interest. In χ^2^ test, Cramer’s V was used to observe the effect size. In the analyses, one category with a socio-economic variable has to be the reference value, and Region Sylhet was chosen (arbitrarily). IBM SPSS 21 (IBM, New York, USA) software was used in data analysis, and the cut-off for significance was *p* < 0.05.

## 3. Results

The occurrence of maternal overnutrition and child undernutrition in the same household was observed in the current study. Of the total sample of overweight mothers, 24.5% had stunted children, 19.8% underweight children, 9.3% wasted children, and 51.7% anaemic children. However, there was significant heterogeneity in the occurrence of the DBM, as can be seen in Table 1.

### 3.1. Overweight Mothers and Stunted Children

**Figure 2:**
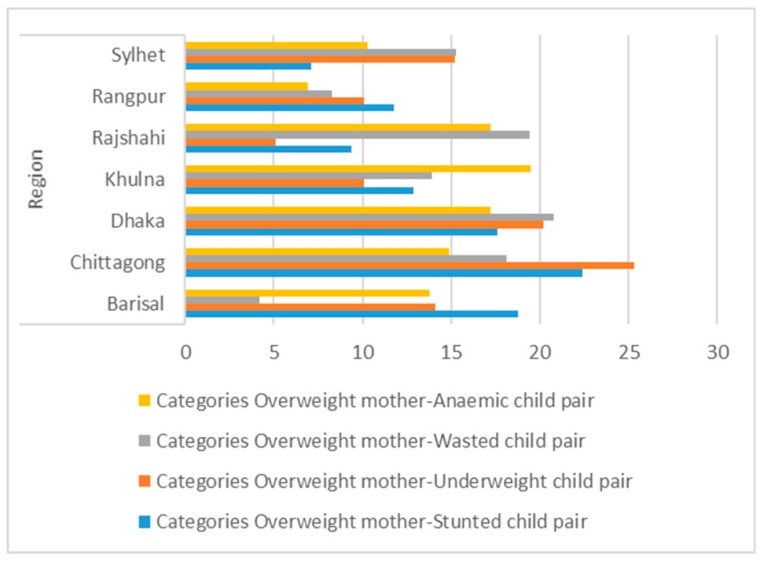
Regional distribution of double burden of malnutrition (DBM).

For overweight and stunting there was marked regional variation (Figure 2) with the highest prevalence in Chittagong (22.4%) and the lowest in Sylhet (7.1%). Rural areas had a higher prevalence (58.8%) while fathers/husbands working in service had a much higher prevalence (44.7%) than other occupations. There was an association with education, and fathers/mothers with primary and secondary education levels had the highest prevalence (>30%).

**Figure 3:**
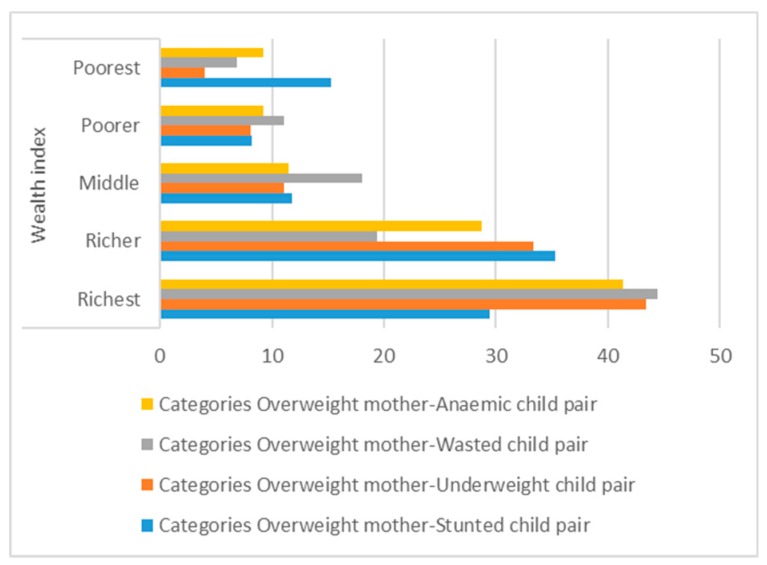
Distribution of DBM across the wealth index.

Households with three possession scores had the highest prevalence (32.9%) as did “richer” households on the wealth index (35.3%) (Figure 3). Households using flush toilets, tube-well water, coal/wood as cooking fuel, and with more than five family members all had higher prevalence.

After sequential multinomial logistic regression, only region, father’s occupation, and wealth index remained significant (*p* < 0.05) Barisal was the region with the highest prevalence after taking into account the other socio-economic variables, while children from richer families on the wealth index had the highest prevalence (Table 2).

### 3.2. Overweight Mothers and Underweight Children

Chittagong had 25% overweight mothers with underweight children. More than half of the overweight mothers with underweight children were found in urban areas. Parental occupation followed a similar trend to the overweight mothers with stunted children. The occurrence of overweight mothers with underweight children increased with improvement of the father’s/husband’s educational status. Overweight mothers with underweight children were more often observed in parents having secondary-level education. The percentages of overweight mothers with underweight children were high in richer families with improved toilet facilities (Table 3). After sequential multinomial logistic regression, only region, father’s occupation, and wealth index remained significant (*p* < 0.05) (Table 4).

### 3.3. Overweight Mothers and Wasted Children

Dhaka had more overweight mothers with wasted children among the seven regions. Urban areas had more overweight mothers with wasted children than rural areas. Parental occupation and type of housing showed a similar trend as those for overweight mothers with stunted/underweight children. The number of overweight mothers with wasted children increased with improvements in parental education, wealth index, possession score, and toilet facilities (Table 5). After sequential multinomial logistic regression, only region and father’s occupation remained significant (*p* < 0.05) (Table 6). The results showed a similar pattern when using the Asian cut-off for BMI to define maternal overweight. In a multinomial regression analysis, father’s/husband’s occupation and wealth index were significantly associated with double burden of malnutrition in mother–child pairs (Appendix A Table A1).

### 3.4. Overweight Mothers and Anaemic Children

Khulna had almost 20% overweight mothers with anaemic children, which was highest among the seven regions. Rural households had a high number of overweight mothers with anaemic children. Fathers working in services had a high number of overweight mothers with anaemic children. More than 80% of the unemployed mothers were overweight with anaemic children.

The number of overweight mothers with anaemic children increased with improvements in father’s education, wealth index, and toilet facilities, and vice versa (Table 7). The results showed a similar pattern when using the Asian cut-off for BMI to define maternal overweight (Appendix A Table A1). In a multinomial regression analysis, region and drinking water were significantly associated with double burden of malnutrition in mother–child pairs. Households having a piped drinking water source were 70% less likely to have underweight mothers with anaemic children (Table 8).

## 4. Discussion

The present paper observed the existence of a double burden of malnutrition in the same household. Overweight mothers with undernourished (stunted/underweight/wasted) children were found under the same roof. When the effects of all other variables were removed, the double burden of malnutrition was significantly associated with region, wealth index, and father’s/husband’s occupation. Overweight mothers and undernourished children were more evident in the richest households (Figure 3). The prevalence of malnourished mother and anaemic children improved with an improvement of drinking water quality. Overweight mothers with anaemic children were 27% more likely to use piped water as the source of their drinking water.

Oddo et al. [18] observed the existence of stunted children and overweight mothers within the same household in Bangladesh. They used data from the Bangladesh Nutritional Surveillance Project (BNSP) (2003–2006) and reported that double-burden households were more likely to be among the wealthier quintiles, which is consistent with the current study result. Other researchers also found similar results [13,23,24]. Although research is limited, an increased prevalence of MCDB has been observed in countries that are in the midst of their nutrition transition [25,26].

In a regional context, Shukla et al. [27] have confirmed the co-existence of under-and overnutrition in South Asian countries. Demographic and socioeconomic transitions have been occurring for the last few decades in this region [28]. Using data collected by Helen Keller International (HKI), Shafique et al. [29] observed an increasing trend of overweight mothers among urban poor women, but it was not widely evident in rural poor women. In the present study, 54% urban and 46.2% rural women were overweight. There are extensive differentials hidden behind these simple urban/rural comparisons. Different socioeconomic groups living in slums within the same urban areas are also exposed to the greatest health risks. The stunting rates among poor urban children are as high as those among poor rural children.

In the present paper, three major big cities (Dhaka, Chittagong, and Khulna) had the highest percentages of overweight mothers with undernourished children (Figure 2). More than 20% of mothers who were overweight had children suffering from either stunting, underweight, or wasting. More than half of the urban households had stunted children with overweight mothers (SCOWT). SCOWT is not entirely an urban phenomenon because the prevalence of SCOWT is affected by both the rates of childhood stunting (high in rural areas) and maternal overweight (high in urban areas) [11].

With increased female employment and related time constraints, urban dwellers also increase their consumption of street foods or processed, ready-to-eat foods. Street foods often contain unhealthy levels of energy, saturated fats, salt, and refined sugars. They are also a good source of microbes responsible for food-borne illnesses due to poor hygiene status [30,31,32]. As a consequence, urban mother–child pairs are prone to both overweight and underweight.

There is a general perception that women living in urban areas are more likely to engage in income-generating activities outside the home than rural women. Previous analysis of the Demographic and Health Surveys in developing countries showed that the main difference between urban and rural areas was not the rate of women’s employment (which was roughly 50% in both areas) but rather the fact that urban mothers were much less likely to take their child to their place of work than rural women [33]. This is likely due to the types of work women engage in: agriculture in rural areas and factory, market, or street work in urban areas, which may be either unsafe or unsuitable for bringing the child along. Given these circumstances, the net impact of urban women’s engagement in paid work on child well-being will depend on the quality of the substitute child care they are able to secure. Little information is available on the type and quality of childcare used by working women and this issue thus remains poorly understood. Evidence suggests, however, that women’s employment and child care decisions are highly influenced by the age of their youngest child [34].

Mothers educated up to primary-level education from poor households with unimproved toilet facility were underweight with anaemic children. Lower rates of extra-household employment and reduced economic power within the household [35,36], higher rates of infection related to poor sanitation or high rates of reproductive tract infections, gynaecological morbidity, or sexually transmitted diseases [37,38] could be the possible causes of mother–child undernutrition. Overweight mothers with anaemic children tended to be from households having at least a possession score of 3 with improved toilet facilities (Table 1). In the South Asian cultural context, the dimensions of autonomy such as freedom of movement, decision-making power, and control over finances can also influence service use and service choice. This may lead to inappropriate treatment of illnesses.

Nutrition transition is caused by globalisation, rapid urbanisation, and economic development. A major shift in food consumption and physical activity patterns is evident in nutrition transition [39]. Urban populations are gradually shifting towards diets containing excessive amounts of energy, saturated fats, refined sugars, and salt, replacing a staple cereal-based diet [39]. Besides that, a sedentary lifestyle in the urban population is increasing the risk of obesity and chronic non-communicable diseases such as coronary heart diseases, diabetes, and cancer.

In recent years, adult overnutrition has come to exist simultaneously with child undernutrition in Bangladesh, which is well known as the double burden of malnutrition. The United Nations Development Programme (UNDP) have confirmed the double burden of malnutrition, i.e., the co-existence of under- and overnutrition, in South Asia. For the last few decades, this region has been undergoing demographic and socioeconomic transitions [40]. These transitions include a shift in nutrition and morbidity patterns, and Bangladesh is gradually paving its way to the double burden of malnutrition. Hence, along with measuring the prevalence of undernutrition, it is obviously important to determine the national prevalence of overweight as well as its determining factors [41].

According to the foetal origin of disease, early (intrauterine or early post-natal) undernutrition causes an irreversible differentiation of metabolic systems. For example, a foetus of an undernourished mother will respond to a reduced energy supply by switching on genes that optimise energy conservation. This survival strategy causes a permanent differentiation of regulatory systems that result in an excess accumulation of energy (and, consequently, of body fat) when the adult is exposed to an unrestricted dietary energy supply. Consequently, an undernourished child grows into an obese adult given the proper environment. Based on our current findings and the limited evidence available to date, it is likely that maternal obesity plays an important role in child anaemia. Kelven et al. observed that the larger size of foetuses of obese mothers may negatively influence cord blood iron markers [42]. Even after adjusting for infant size at birth, maternal obesity and excessive gestational weight gain were negatively associated with neonatal iron status. However, the mechanisms underlying this association are still unclear [43,44,45].

Increasing economic status and gross domestic product (GDP) decrease undernutrition but the relationship between GDP and nutrition is complex. Being poor in a middle-income country increases the risk of obesity compared with being rich in the same country [37]. In urbanised developing countries, the availability of cheap, energy-dense foods could be the hidden cause of obesity. Besides that, undernutrition in children (aged under five years) tends to increase in urban areas of countries in socio-economic transition [39]. Caballero [44] defined this phenomenon as the “underweight–overweight paradox”. Effectively, the underweight–overweight paradox poses a challenge to public health programs, since most programmes are designed to reduce undernutrition. Large-scale nutrition-sensitive programmes which address the key underlying determinants of nutrition are necessary to enhance the coverage and effectiveness of nutrition-specific interventions [45].

## 5. Conclusions

The increasing number of overweight mothers is becoming a new health concern for Bangladesh. The results of this recent study clearly show that double burden exists in Bangladesh and in the same household. Apart from that, the co-existence of overweight mother and undernourished children in the same household portrays the complex dynamics of possible causes. Future longitudinal studies are needed to understand the factors linked to DBM risk. The interaction between maternal overweight/obesity and early childhood undernutrition can be identified and addressed by effective large-scale population studies to reduce the long-term risk of obesity in the offspring.

## 6. Limitations

The Bangladesh Demographic and Health Survey is based on a cross-sectional study design. Although the data collection and study design were of high quality, the data did not allow us to draw causal relationships between variables in the current study. The data lacked information on maternal energy intake, physical activity level, and micronutrient intake, which are important for a proper assessment of nutritional status. Only one in every three households was selected for haemoglobin concentration assessment, which may not give a clear idea about the anaemia status of the whole population. It was not possible to examine the relationship between child feeding practise and infection and nutritional status due to a large amount of missing information on complementary feeding, immunisation, and infectious disease. Incomplete information and inconsistent information on some selected variables complicated the analysis process considerably.

## Figures and Tables

**Table 1:** Prevalence of overweight mother and stunted child pairs through the socio-demographic variables.

Variables	Categories	OvM & StC		χ²	*p*-Value	CV	Effect Size
		*N*	%				
Region	Barisal	16	18.8	84.3	<0.001	0.1	Small
	Chittagong	19	22.4				
	Dhaka	15	17.6				
	Khulna	11	12.9				
	Rajshahi	8	9.4				
	Rangpur	10	11.8				
	Sylhet	6	7.1				
Location	Urban	35	41.2	130.5	<0.001	0.2	Small
	Rural	50	58.8				
Father’s occupation	Service	38	44.7	234.1	<0.001	0.1	Small
	Skilled manual	18	21.2				
	Unskilled labour	10	11.8				
	Agricultural work	16	18.8				
	Unemployed	3	3.5				
Mother’s occupation	Service	5	5.9	71.2	<0.001	0.1	Small
	Skilled manual	0	0				
	Unskilled labour	2	2.4				
	Agricultural work	5	5.9				
	Unemployed	73	85.9				
Father’s education	Higher	18	21.2	282.2	<0.001	0.2	Small
	Secondary	26	30.6				
	Primary	26	30.6				
	No education	15	17.6				
Mother’s education	Higher	11	12.9	236.1	<0.001	0.2	Small
	Secondary	40	47.1				
	Primary	26	30.6				
	No education	8	9.4				
Possession score	≥4	11	12.9	310.1	<0.001	0.2	Small
	3	28	32.9				
	2	20	23.5				
	1	10	11.8				
	0	16	18.8				
Wealth index	Richest	25	29.4	489.5	<0.001	0.2	Small
	Richer	30	35.3				
	Middle	10	11.8				
	Poorer	7	8.2				
	Poorest	13	15.3				
Source of drinking water	Pipe	12	14.1	148.9	<0.001	0.1	Small
	Tube well	71	83.5				
	Rest	2	2.4				
	Total	85	100				
Toilet facility	Flush	30	35.3	278.8	<0.001	0.2	Small
	Pit with slab	28	32.9				
	Unimproved	27	31.8				
Cooking fuel	Gas/Electricity	16	18.8	260	<0.001	0.2	Small
	Coal/Wood	49	57.6				
	Agricultural crops	20	23.5				
House type	All brick/Brick wall	33	38.8	244.9	<0.001	0.2	Small
	All tin	45	52.9				
	Tin roof	5	5.9				
	All thatch	2	2.4				
Number of household members	1 to 4	24	28.2	3.2	<0.001	0.03	Small
	≥5	61	71.8				

StC = stunted child; OvM = overweight mother; CV = Cramer’s V.

**Table 2:** Sequential multinomial logistic regression analysis of overweight mother and stunted child pairs by socio-demographic variables.

DBM	Variables	Categories	OR	CI	χ^2^	*p*-Value	*R* ^2^	Effect Size
Overweight mother	Region	Barisal	3.79	2.81–4.78	64.9	0.004	0.3	Small
with stunted child		Chittagong	1.87	1.41–2.33				
		Dhaka	1.7	1.31–2.10				
		Khulna	1.74	1.33–2.15				
		Rajshahi	1.4	1.15–1.65				
		Rangpur	1.8	1.37–2.24				
		Sylhet	1	-				
	Wealth index	Richest	0.4	0.68–0.92	48.1	<0.001	0.3	Small
		Richer	0.69	0.49–0.87				
		Middle	0.35	0.42–0.88				
		Poorer	0.36	0.41–0.88				
		Poorest	1	-				

OR = odds ratio; CI = confidence interval.

**Table 3:** Prevalence of overweight mothers and underweight children through socio-demographic variables.

Variables	Categories	OvM & UnC		χ²	*p*-Value	CV	Effect Size
		*N*	%				
Region	Barisal	14	14.1	99.6	<0.001	0.1	Small
	Chittagong	25	25.3				
	Dhaka	20	20.2				
	Khulna	10	10.1				
	Rajshahi	5	5.1				
	Rangpur	10	10.1				
	Sylhet	15	15.2				
Location	Urban	55	55.6	224.7	<0.001	0.2	Small
	Rural	44	44.4				
Father’s occupation	Service	46	46.5	353	<0.001	0.2	Small
	Skilled manual	19	19.2				
	Unskilled labour	15	15.2				
	Agricultural work	16	16.2				
	Unemployed	3	3				
Mother’s occupation	Service	1	1	92.4	<0.001	0.1	Small
	Skilled manual	2	2				
	Unskilled labour	3	3				
	Agricultural work	2	2				
	Unemployed	91	91.9				
Father’s education	Higher	20	20.2	464.9	<0.001	0.2	Small
	Secondary	39	39.4				
	Primary	19	19.2				
	No education	21	21.2				
Mother’s education	Higher	9	9.1	413.9	<0.001	0.2	Small
	Secondary	42	42.4				
	Primary	26	26.3				
	No education	22	22.2				
Possession score	≥4	17	17.2	604.7	<0.001	0.2	Small
	3	27	27.3				
	2	35	35.4				
	1	10	10.1				
	0	10	10.1				
Wealth index	Richest	43	43.4	814.5	<0.001	0.2	Small
	Richer	33	33.3				
	Middle	11	11.1				
	Poorer	8	8.1				
	Poorest	4	4				
Source of drinking water	Pipe	20	20.2	213.7	<0.001	0.2	Small
	Tube well	79	79.8				
	Rest	0	0				
Toilet facility	Flush	56	56.6	469.3	<0.001	0.2	Small
	Pit with slab	24	24.2				
	Unimproved	19	19.2				
Cooking fuel	Gas/Electricity	22	22.2	356.7	<0.001	0.2	Small
	Coal/Wood	62	62.6				
	Agricultural crops	15	15.2				
House type	All brick/Brick wall	51	51.5	436.3	<0.001	0.2	Small
	All tin	39	39.4				
	Tin roof	9	9.1				
	All thatch	0	0				
Number of household members	1 to 4	32	32.3	3.3	ns	0.03	Small
	≥5	67	67.7				

UnC = underweight child; OvM = overweight mother; CV = Cramer’s V.

**Table 4:** Sequential multinomial logistic regression analysis of overweight mother and underweight child pairs by socio-demographic variables.

DBM	Variables	Categories	OR	CI	χ^2^	*p*-Value	*R* ^2^	Effect Size
Overweight mother	Region	Barisal	0.92	0.88–0.96	68.9	<0.001	0.3	Small
with underweight child		Chittagong	0.87	0.80–0.94				
		Dhaka	0.73	0.56–0.89				
		Khulna	0.45	0.42–0.88				
		Rajshahi	0.31	0.35–0.87				
		Rangpur	0.47	0.46–0.88				
		Sylhet	1	-				
	Father’s occupation	Service	1.33	1.14–1.53	40.5	0.002	0.3	Small
		Skilled manual	0.86	0.76–0.96				
		Unskilled labour	1.04	1.01–1.07				
		Agricultural work	1.05	1.02–1.08				
		Unemployed	1	-				
	Wealth index	Richest	1.04	1.02–1.06	53.1	<0.001	0.3	Small
		Richer	0.93	0.89–0.97				
		Middle	0.82	0.72–0.93				
		Poorer	1.18	1.10–2.10				
		Poorest	1	-				

**Table 5:** Prevalence of overweight mother and wasted child pairs through the socio-demographic variables.

Variables	Categories	OvM & WaC		χ^2^	*p*-Value	CV	Effect Size
		*N*	%				
Region	Barisal	3	4.2	74.7	<0.001	0.1	Small
	Chittagong	13	18.1				
	Dhaka	15	20.8				
	Khulna	10	13.9				
	Rajshahi	14	19.4				
	Rangpur	6	8.3				
	Sylhet	11	15.3				
Location	Urban	39	54.2	163	<0.001	0.2	Small
	Rural	33	45.8				
Father’s occupation	Service	44	61.1	304.8	<0.001	0.1	Small
	Skilled manual	10	13.9				
	Unskilled labour	6	8.3				
	Agricultural work	10	13.9				
	Unemployed	2	2.8				
Mother’s occupation	Service	4	5.6	75.3	<0.001	0.1	Small
	Skilled manual	2	2.8				
	Unskilled labour	2	2.8				
	Agricultural work	2	2.8				
	Unemployed	62	86.1				
Father’s education	Higher	27	37.5	352.7	<0.001	0.2	Small
	Secondary	20	27.8				
	Primary	19	26.4				
	No education	6	8.3				
Mother’s education	Higher	15	20.8	331.4	<0.001	0.2	Small
	Secondary	31	43.1				
	Primary	21	29.2				
	No education	5	6.9				
Possession score	≥4	17	23.6	422.7	<0.001	0.2	Small
	3	20	27.8				
	2	14	19.4				
	1	11	15.3				
	0	10	13.9				
Wealth index	Richest	32	44.4	569.1	<0.001	0.2	Small
	Richer	14	19.4				
	Middle	13	18.1				
	Poorer	8	11.1				
	Poorest	5	6.9				
Source of drinking water	Pipe	23	31.9	190.1	<0.001	0.2	Small
	Tube well	48	66.7				
	Rest	1	1.4				
Toilet facility	Flush	39	54.2	350.9	<0.001	0.2	Small
	Pit with slab	16	22.2				
	Unimproved	17	23.6				
Cooking fuel	Gas/Electricity	26	36.1	288.9	<0.001	0.2	Small
	Coal/Wood	33	45.8				
	Agricultural crops	13	18.1				
House type	All brick/Brick wall	36	50	283.9	<0.001	0.2	Small
	All tin	31	43.1				
	Tin roof	5	6.9				
	All thatch	0	0				
Number of household members	1 to 4	26	36.1	2.9	ns	0.3	Small
	≥5	46	63.9				

WaC = wasted child; OvM = overweight mother; CV = Cramer’s V.

**Table 6:** Sequential multinomial logistic regression analysis of overweight mother and wasted child pairs by socio-demographic variables.

DBM	Variables	Categories	OR	CI	χ^2^	*p*-Value	*R* ^2^	Effect Size
Overweight mother	Region	Barisal	0.41	0.30–0.92	60.7	0.001	0.3	Small
with wasted child		Chittagong	0.82	0.70–0.95				
		Dhaka	0.9	0.83–0.97				
		Khulna	1.43	1.20–1.66				
		Rajshahi	1.72	1.38–2.06				
		Rangpur	0.79	0.64–0.94				
		Sylhet	1	-				
	Father’s occupation	Service	1.15	1.10–1.25	42.7	0.002	0.3	Small
		Skilled manual	0.45	0.36–0.94				
		Unskilled labour	0.43	0.31–0.95				
		Agricultural work	0.38	0.42–0.94				
		Unemployed	1	-				

**Table 7:** Prevalence of overweight mother and anaemic child pairs by socio-demographic variables.

Variables	Categories	OvM & AC		χ²	*p*-Value	CV	Effect Size
		*N*	%				
Region	Barisal	12	13.8	54.7	<0.001	0.1	Small
	Chittagong	13	14.9				
	Dhaka	15	17.2				
	Khulna	17	19.5				
	Rajshahi	15	17.2				
	Rangpur	6	6.9				
	Sylhet	9	10.3				
Location	Urban	36	41.4	101.3	<0.001	0.2	Small
	Rural	51	58.6				
Father’s occupation	Service	38	43.7	95.9	<0.001	0.1	Small
	Skilled manual	18	20.7				
	Unskilled labour	12	13.8				
	Agricultural work	15	17.2				
	Unemployed	4	4.6				
Mother’s occupation	Service	10	11.5	44.5	<0.001	0.1	Small
	Skilled manual	1	1.1				
	Unskilled labour	0	0				
	Agricultural work	2	2.3				
	Unemployed	74	85.1				
Father’s education	Higher	19	21.8	102.6	<0.001	0.2	Small
	Secondary	33	37.9				
	Primary	25	28.7				
	No education	10	11.5				
Mother’s education	Higher	16	18.4	82.1	<0.001	0.2	Small
	Secondary	41	47.1				
	Primary	24	27.6				
	No education	6	6.9				
Possession score	≥4	11	12.6	200.1	<0.001	0.2	Small
	3	29	33.3				
	2	21	24.1				
	1	11	12.6				
	0	15	17.2				
Wealth index	Richest	36	41.4	263.6	<0.001	0.2	Small
	Richer	25	28.7				
	Middle	10	11.5				
	Poorer	8	9.2				
	Poorest	8	9.2				
Source of drinking water	Pipe	17	19.5	107.5	<0.001	0.2	Small
	Tube well	67	77				
	Rest	3	3.4				
Toilet facility	Flush	47	54	149.9	<0.001	0.2	Small
	Pit with slab	21	24.1				
	Unimproved	19	21.8				
Cooking fuel	Gas/Electricity	17	19.5	132.8	<0.001	0.2	Small
	Coal/Wood	53	60.9				
	Agricultural crops	17	19.5				
House type	All brick/Brick wall	44	50.6	125.3	<0.001	0.2	Small
	All tin	38	43.7				
	Tin roof	4	4.6				
	All thatch	1	1.1				
Number of household members	1 to 4	26	29.9	4	ns	0.3	Small
	≥5	61	70.1				

AC = Anaemic child; OvM = overweight mother; CV = Cramer’s V.

**Table 8:** Sequential multinomial logistic regression analysis of overweight mother and anaemic child pairs by socio-demographic variables.

DBM	Variables	Categories	OR	CI	χ²	*p*-Value	*R*²	Effect Size
Overweight mother	Region	Barisal	3.13	2.32–3.94	55.1	0.003	0.4	Small
with anaemic child		Chittagong	0.92	0.86–0.98				
		Dhaka	1.29	1.11–1.47				
		Khulna	2.89	2.11–3.63				
		Rajshahi	2.68	1.97–3.39				
		Rangpur	1.26	1.09–1.43				
		Sylhet	1	-				
	Drinking water	Pipe	0.73	0.50–0.96	26.9	0.003	0.4	Small
		Tube well	0.65	0.34–0.95				
		Rest	1	-				

## Appendix A

**Table A1:** Sequential multinomial logistic regression analysis of DBM in mother–child pairs by socio-demographic variables for the Asian cut-off point (maternal BMI).

DBM	Variables	Categories	OR	CI	χ^2^	*p*-Value	*R* ^2^	Effect Size
Overweight mother with stunted child	Region	Barisal	1.6	1.25–1.95	54.5	0.004	0.3	Small
		Chittagong	1.15	1.05–1.25				
		Dhaka	1.19	1.06–1.31				
		Khulna	0.87	0.77–0.97				
		Rajshahi	0.68	0.40–0.97				
		Rangpur	1.39	1.15–1.63				
		Sylhet	1	-				
	Father’s occupation	Service	1.01	1.00–1.02	41.1	0.004	0.3	Small
		Skilled manual	0.7	0.57–0.83				
		Unskilled labour	0.61	0.24–0.97				
		Agricultural work	0.7	0.44–0.96				
		Unemployed	1	-				
	Wealth index	Richest	0.67	0.46–0.88	54.1	<0.001	0.3	Small
		Richer	1.13	1.11–1.19				
		Middle	0.64	0.41–0.87				
		Poorer	1.05	1.02–1.08				
		Poorest	1	-				
Overweight mother with underweight child	Region	Barisal	0.92	0.88–0.96	64.2	<0.001	0.3	Small
		Chittagong	0.87	0.86–0.88				
		Dhaka	0.73	0.56–0.90				
		Khulna	0.45	0.03–0.87				
		Rajshahi	0.61	0.35–0.87				
		Rangpur	0.56	0.25–0.86				
		Sylhet	1	-				
	Father’s occupation	Service	1.33	1.14–1.52	42.8	0.002	0.3	Small
		Skilled manual	0.86	0.76–0.86				
		Unskilled labour	1.04	1.01–1.17				
		Agricultural work	1.05	1.02–1.08				
		Unemployed	1	-				
	Wealth index	Richest	1.04	1.02–1.06	60.2	<0.001	0.3	Small
		Richer	0.93	0.89–0.97				
		Middle	0.82	0.72–0.92				
		Poorer	1.18	1.09–1.27				
		Poorest	1	-				
Overweight mother with wasted child	Father’s occupation	Service	1.06	1.02–1.10	42.7	0.002	0.3	Small
		Skilled manual	0.96	0.93–0.99				
		Unskilled labour	1.28	1.11–1.45				
		Agricultural work	1.43	1.19–1.67				
		Unemployed	1	-				
	Wealth index	Richest	0.57	0.28–0.86	53.1	<0.001	0.3	Small
		Richer	1.01	1.00–1.02				
		Middle	1.68	0.75–1.95				
		Poorer	1.54	1.31–1.77				
		Poorest	1	-				
Overweight mother with anaemic child	Region	Barisal	3.13	2.32–3.94	55.1	0.003	0.4	Small
		Chittagong	0.92	0.86–0.98				
		Dhaka	1.29	1.11–1.47				
		Khulna	2.89	2.11–3.63				
		Rajshahi	2.68	1.97–3.39				
		Rangpur	1.26	1.09–1.43				
		Sylhet	1	-				
	Drinking water	Pipe	0.73	0.50–0.96	26.9	0.003	0.4	Small
		Tube well	0.65	0.34–0.95				
		Rest	1	-				
